# C-751-03. Evaluation of Developing Lymphedema in Post-burn Patients Using Lymphatic Mapping

**DOI:** 10.1093/jbcr/irag033.008

**Published:** 2026-04-06

**Authors:** Stephanie M Bond, Mark Fisher, Damon Cooney

**Affiliations:** Johns Hopkins Adult Burn Center, Baltimore, MD; Johns Hopkins Adult Burn Center, Baltimore, MD; Johns Hopkins Adult Burn Center, Baltimore, MD

## Abstract

**Introduction:**

Burns cause complex injuries to many tissues, as well as to limb perfusion. One system that has received little attention so far is the lymphatic. Advances in the field of lymphatic surgery has created new understanding of both the physiologic evaluation of the lymphatic system and created new therapies, and we wonder how these may be helpful in burn reconstruction. The relationship between burn injury and the development of lymphedema has thus far been poorly defined, though circumferential extremity involvement and fascial excision have been identified as risks for the development of the lymphedema disease process that are frequently present in the burned patient population. Lymphatic function was evaluated in patients with these burn characteristics to study the relationship of burn injuries and lymphatic dysfunction.

**Methods:**

The presence of lymphedema was assessed in a prospective study of patients presenting to burn clinic. The characteristics of circumferential burn injury and fascial excision with skin grafting were utilized in the selection of patients at risk for developing lymphedema for lymphatic evaluation. Indocyanine green (ICG) near-infrared lymphatic mapping was performed in two selected patients with circumferential upper extremity burns to assess their lymphatic function. ICG (0.1 cc per injection site) was administered intradermally at the first and fourth web spaces and medial and lateral proximal forearm, and imaging was conducted using a portable handheld imaging system.

**Results:**

Lymphatic channels were successfully visualized in both patients without evidence of dermal backflow within burn tissue. In one patient with a forearm skin graft, lymphatic channels appeared significantly deeper in the grafted region than normal, although no dysfunction was identified. These patients had been identified as having post-burn distal extremity swelling in the setting of burn characteristics concerning for the development of lymphedema, without previous imaging-confirmed lymphedema.

**Conclusions:**

These findings suggest resilience in the lymphatic system despite burn injury, with altered channel depth in areas of prior grafting. The role of the lymphatic system in the evolving story of chronic swelling in the burn patient is still unclear. Further observations to elucidate the response of the lymphatic system in managing swelling are warranted.

**Applicability of Research to Practice:**

This study clarifies the relationship of burn injuries and lymphatic function, suggesting that these patients are not at inherent risk of developing this disease process, and at this stage would likely not benefit from prophylactic therapies.

**Funding for the study:**

N/A.

